# Erythema nodosum leprosum presenting as pyrexia of unknown origin

**DOI:** 10.1186/1471-2334-14-S3-P69

**Published:** 2014-05-27

**Authors:** Aravind Reghukumar, Athul Gurudas, VS Kiran Kumar, Ranjani Ravi

**Affiliations:** 1 Department of Infectious Diseases, Medical College Hospital, Thiruvananthapuram, India; 2 Cosmopolitan Hospital, Thiruvananthapuram, India

## Background

Erythema nodosum leprosum (ENL) or Type 2 lepra reaction is an immune complex mediated hypersensitivity reaction. We report a rare scenario where ENL presented as pyrexia of unknown origin (PUO).

## Case report

A 63 year old lady presented to a peripheral centre polyarthralgia, multiple tender and erythematous nodules over her face, elbows, back and legs and continuous fever of 2 months duration. Routine investigations being negative, she was referred for further evaluation of PUO. On presentation, the patient was febrile. Apart from the nodules, examination revealed bilaterally thickened and tender greater auricular, ulnar and common peroneal nerves. Her left hand was swollen and tender due to ulnar neuritis. Bilateral symmetric sensory neuropathy was present upto the ankles. Few of the lesions over the face were found to be hypoaesthetic. Laboratory investigations showed mild transaminitis and an ESR of 64. Biopsy of a nodule showed septal panniculitis suggestive of erythema nodosum. ANA profile, cANCA and pANCA were negative. Split skin smears were positive for acid fast bacilli (AFB) with bacillary index between 2+ to 4+. Skin biopsy showed ill defined granuloma formation with occasional multinucleate giant cells. Fite’s stain revealed numerous AFB. A diagnosis of multibacillary leprosy with ENL was made. She was treated with prednisolone 1mg/kg/day followed by multidrug therapy comprising of dapsone, clofazimine and rifampicin. fever, neuritis and polyarthritis dramatically subsided after steroid initiation. Steroids were tapered and stopped over the next 2 months. This case is being presented because of rarity of presentation of ENL as PUO.

